# Real-Time Classification of Diesel Marine Engine Loads Using Machine Learning

**DOI:** 10.3390/s19143172

**Published:** 2019-07-18

**Authors:** Syed Maaz Shahid, Sunghoon Ko, Sungoh Kwon

**Affiliations:** 1 School of Electrical Engineering, University of Ulsan, Ulsan 44610, Korea; 2 Hyundai Heavy Industries, Ulsan 44032, Korea

**Keywords:** diesel engine, cylinder banks, load classification, real measured data, crank angle, neural network, machine learning

## Abstract

An engine control system is responsible for controlling the combustion parameters of an internal combustion engine to increase the efficiency of the engine. An optimized parameter setting of an engine control system is highly influenced by the engine load. Therefore, with a change in engine load, the parameter settings need to be updated for higher engine efficiency. Hence, to optimize parameter settings during operation, engine load information is necessary. In this paper, we propose a real-time engine load classification from sensed signals. For the classification, an artificial neural network is used and trained using processed, real, measured data. To that end, a magnetic pickup sensor extracts the rotational speed of the prime mover of a four-stroke V12 marine diesel engine. The measured signal is then converted into a crank angle degree (CAD) signal that shows the behavior of the combustion strokes of firing cylinders at a particular engine load. The CAD signals are considered an input feature to the designed network for classification of engine loads. For verification, we considered five classes of engine load, and the trained network classifies these classes with an accuracy of 99.4%.

## 1. Introduction

Diesel marine engines are used in marine vehicles for main propulsion and provide auxiliary power to vessels [1]. Marine engines are internal combustion (IC) engines that generate thermal energy by burning fuel and converting it into mechanical energy. However, exposed to a harsh environment, marine diesel engines are prone to breakdown [2], leading to maritime disasters. To secure and enhance the reliability of the marine engine, it is imperative to monitor the engine condition. For that purpose, monitoring systems are installed, and a maintenance strategy called condition-based monitoring (CBM) [3] continually monitor the status of an engine in real time using monitoring systems.

For CBM, different sensors are used to monitor engine operation. Sensors can be chosen according to the method used to analyze the internal combustion process of an engine. Engine cylinder pressure is the most direct parameter for observing the internal combustion of a diesel engine. However, the problem with this method is that a pressure sensor has difficulty working over the long term and is expensive [4]. An alternative approach is vibration measurement [5], which is widely used in the manufacturing industry since vibration is an intrinsic mechanical phenomenon. The vibration measured from the cylinder head contains a lot of effective information about the combustion process [6]. Another approach to monitor the engine condition is to analyze torque in the engine system. Internal combustion in an engine is the combustion strokes of the firing cylinders, which produce the mechanical energy. The mechanical energy generates the tangential force that increases the rotational speed of mechanical components of the engine, and the rotational speed of the components fluctuates based on the effect of the combustion strokes in the firing cylinders. By monitoring the fluctuation of the rotational speed of the moving parts of the engine, we can analyze the internal combustion process that shows the status of engine operation.

Magnetic pickup (MPU) sensors are used to detect the rotation speed of a prime mover of an engine. MPU sensors are electromagnetic devices that sense a sudden change in a magnetic field and use as a tachometer. As each tooth of a gear passes the magnetic field of an MPU sensor, it interrupts the magnetic field, and an alternating voltage is created by the MPU sensor. The frequency of this voltage is translated by the tachometer that accurately depicts the speed of the prime mover. By analyzing the change in the voltage signal developed by the MPU sensor, we can observe the behavior of the combustion strokes of the firing cylinders in the engine. The behavior of the engine depends on the imposed load, referred to as engine load, and the behavior of the engine refers to the combustion strokes of the firing cylinders in the engine. The engine load is the power of the engine, i.e., the torque output of the engine, and it affects the combustion parameters of an engine. An electronic control engine has many combustion parameters, such as air-fuel ratio, exhaust temperature, and torque [7], which affect the efficiency of the engine. Based on the engine load conditions, the parameters of an IC engine need to be optimized to increase the efficiency of the engine. Improvement in efficiency yields significant benefits in fuel consumption and lessens exhaust emissions [8]. Knowing engine load information during operation helps to adjust the parameters, since optimum parameters depend on the engine load.

To increase the efficiency of an operating engine, researchers have proposed many techniques and methods to optimize parameters of IC diesel engines. In [9], the authors proposed a method to adjust the fuel injection parameters to improve the efficiency of the engine using a generic algorithm. In [10], intake and fuel control parameters were optimized using regression models. Because different engine loads have different optimal setting for parameters, knowing engine load information during operation helps to adjust parameters. Although researchers did not consider any mechanism to get exact engine load information to determine optimized settings for the parameters, they have proposed many schemes to tackle the problem of fault detection and classification of the diesel engine to increase the reliability of engine operation. In [11], the authors proposed the fault classification scheme based on the audio signature of an engine. The proposed scheme used Fourier transform and correlation methods for classification between healthy and faulty classes. A magnetic head-type torque sensor was adopted in [12] to detect engine misfires. Sensor signal treatment using fast Fourier transformation was used to detect misfiring cylinders at high rotational speeds. Artificial intelligence (AI)-based techniques have also been studied for fault detection and classification of a diesel engine, as well for optimizing parameters of the diesel engine. In [13], an intelligent fault diagnostic technique was proposed for marine diesel engines. In this technique, instantaneous angular speed signals are used in conjunction with a support vector machine to assess the health of diesel engines. A multilayer perceptron neural network (MLPNN) was used for fault classification of an IC engine [14]. The vibration signals were pre-processed by wavelet analysis, and signal energy was considered a distinguishing property to classify faults. An artificial neural network (ANN)-based method was proposed for fault detection in a marine diesel engine [15].

However, previous studies did not consider the engine load classification, which would be useful in improving engine efficiency while the engine is operating [16]. The reliability of the engine can be improved by adjusting the parameters of the engine using engine load information. Therefore, load classification is an important tool in advanced engine control systems to increase engine efficiency. To the best of our knowledge, no previous work addresses the problem of engine load classification. The results of this study can be used as a safety method to detect cylinder misfires. Engine load classification is similar to engine fault classification because we must analyze the status of engine operation to extract characteristics in both cases. Since an engine misfire is generally detected using reduced exhaust gas temperature, it takes dozens of seconds to detect a misfire. Furthermore, this method can be used to detect misfire immediately, preventing the risk of engine damage due to misfires.

We propose a real-time engine load-classification method using a machine learning classifier. Machine learning mostly uses two types of techniques to train a classifier [17]: supervised learning, which trains a model based on known input/output pairs (so it can predict future output), and unsupervised learning, which identifies patterns in the input data when the input data are not labeled. An ANN classifier is used along with supervised learning since the ANN is a good tool to account for dynamic transitions and the nonlinear behaviors of the engine, and it is easily configurable when passing from one engine to another [15]. The feedforward neural network is the type of ANN used for classification in this research. To learn the distinct features of different engine loads, supervised learning is used to train the network. According to the features learned in training using measured signals from actual operating engine, the network can classify the load on the engine. A magnetic pickup sensor is used to compare engine operation at different loads. Time series sensor data is converted to crank angle degree (CAD) signals, which show the ignition behavior of the firing cylinders at a particular engine load. Therefore, CAD signals are used as a feature vector, and are fed into the designed network to classify engine loads. The designed network is capable of classifying engine loads with an accuracy of more than 99%. We also compared the classification results of the designed network with the multi-class support vector machine (SVM), which is another supervised learning model used for classification [18]. Compared to earlier work [19], this paper provides more details on pre-processing of sensor signals, uses a better algorithm to train the designed classifier, and verifies the classifier through more engine load data sets.

The remaining of the paper is organized as follows. Section 2 presents the details of the V-type engine with multiple cylinder banks, and Section 3 explains the proposed method to classify engine loads. Section 4 presents an experimental data acquisition. Section 5 and Section 6 describe the pre-processing sensor data and the designed ANN, respectively. Section 7 shows the load-classification efficiency with the designed ANN. Section 8 concludes this paper.

## 2. Cylinder Banks

**Figure 1:**
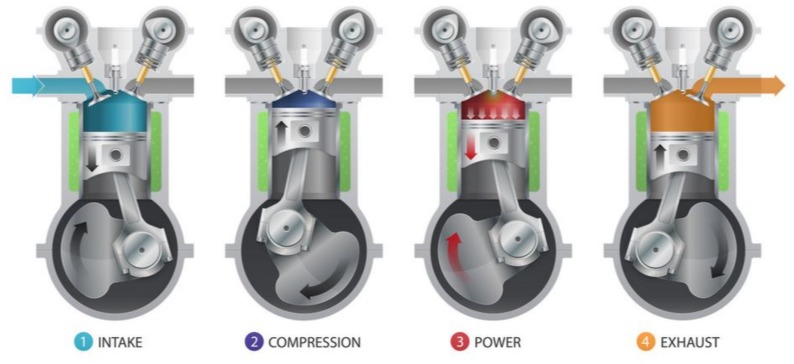
Four-stroke cycle engine [20].

A cylinder is the central working part of an engine and chamber where the piston travels. In four-stroke engines, the piston moves to the topmost point called top dead center (TDC), and to the bottommost point of the cylinder (BDC), called bottom dead center, two times, and the crankshaft completes two revolutions over four piston cycles. Another common design for an engine (based on the stroke type) is the two-stroke engine in which the piston moves to TDC and to BDC once, and the crankshaft completes one revolution during two piston strokes. Figure 1 shows a four-stroke engine (intake, compression, combustion [or power], and exhaust) in which the crankshaft completes two revolutions. On intake and combustion, the piston moves to BDC whereas during compression and exhaust, the piston moves to TDC. The pistons transform the energy of the expanding gas into mechanical energy.

**Figure 2:**
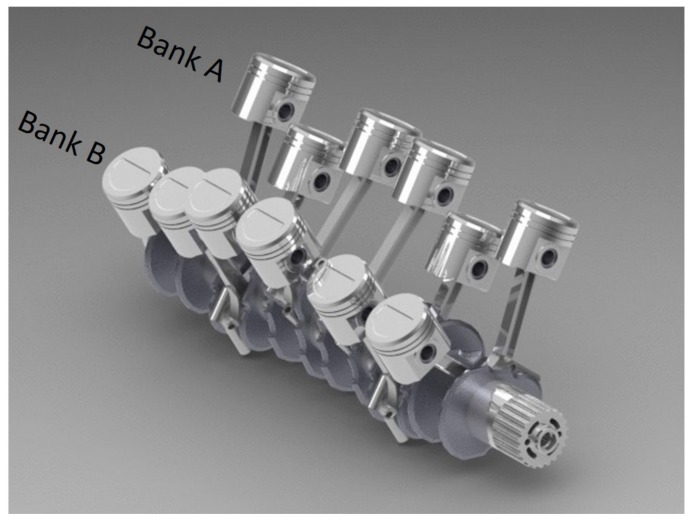
V12 engine: 12 cylinders are arranged in two banks of six cylinders set at an angle to one another [21].

In multi-cylinder IC engines, cylinders are typically arranged in one of three ways: in-line, flat, or a V shape. For the in-line engine, all cylinders are in a row, whereas a flat engine has two lines of cylinders that are directly opposite to each other. The V-type engine also has two lines of cylinders, but they are placed at an angle to each other. Each line of cylinders is referred to as a cylinder bank, and the angle between the cylinder banks is known as the bank angle. An advantage of placing cylinders in banks is that the engine is shorter in length. Two cylinder banks of a V12 engine, referred to as Bank A and Bank B, are shown in Figure 2, and each bank of the V12 engine has six cylinders. Each bank has a particular firing sequence, which is the combustion sequence of the cylinder, or power delivery, in a multi-cylinder engine.

## 3. Proposed Method for Classification of Engine Loads

**Figure 3:**
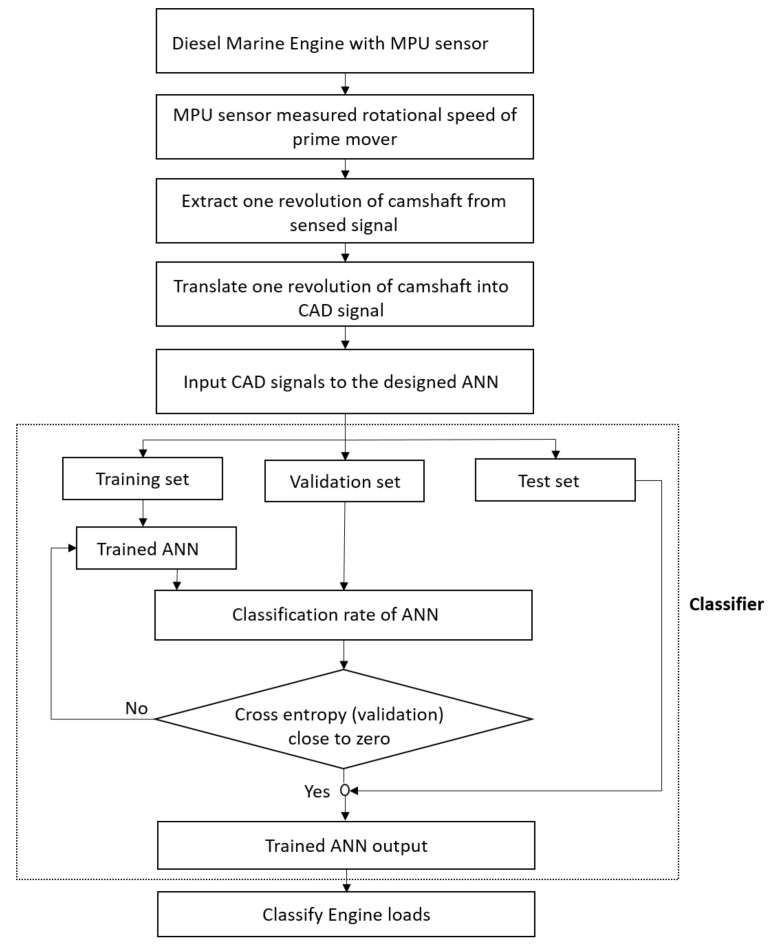
Flow of the engine load-classification algorithm.

To get the load information of a marine diesel engine, we introduce an engine load-classification method using an ANN. The proposed method is a three-step classification algorithm. First, the algorithm extracts single-period signals from the raw signals of an MPU sensor. A single period represents one revolution of a camshaft in which combustion strokes of 12 cylinders in the engine are completed. Based on that, firing the 12 cylinders in the internal combustion process then translates into a CAD signal. In the CAD signal, the effect of the combustion stroke of each firing cylinder can be observed at a particular engine load. At different engine loads, the effects of combustion strokes of firing cylinder are different and are analyzed using the CAD signals. In the end, the CAD signals are fed into the designed classifier, i.e., an ANN, and the ANN is trained using supervised learning. The CAD signals are fed to the ANN as input signals for training, and after training, the trained network is used for real-time classification of engine loads. Real-time classification enables us to get the engine load information for each revolution of the camshaft. The flow of the proposed method is shown in Figure 3. A detailed explanation of each step is presented in the next sections.

## 4. Experimental Data Acquisition

**Figure 4:**
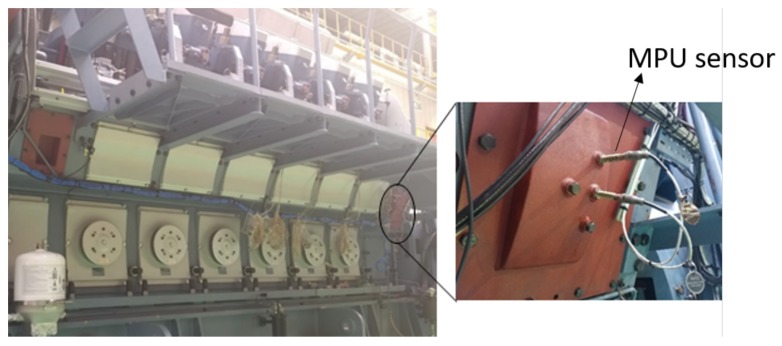
Four-stroke marine diesel engine with the location of the pickup sensor.

**Figure 5:**
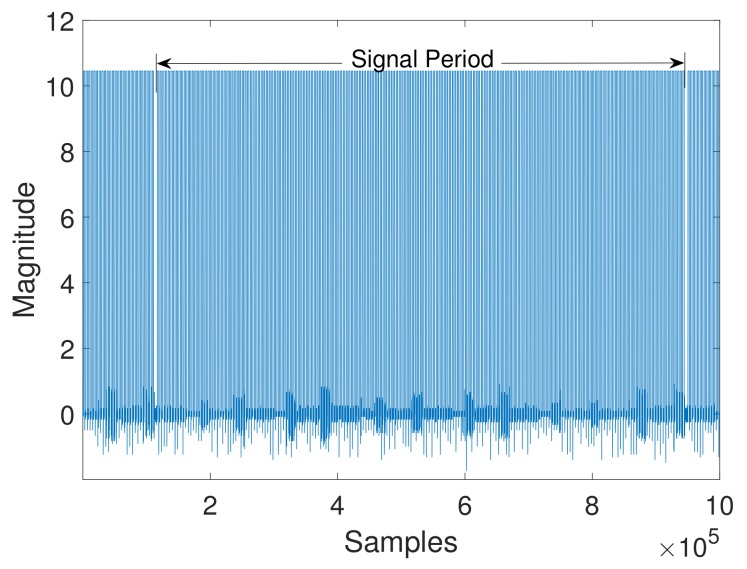
Pickup sensor data.

Data acquisition from an engine is a process of measuring the dynamic parameters of the engine. For data acquisition, a V12 four-stroke IC marine diesel engine was used and tested at different loads. The experimental tests of this research were done in collaboration with the R&D center of Hyundai Heavy Industries. The diesel engine provides auxiliary power to a marine vessel. The engine ran at 720 rpm, and the load varied from 0% to 100%. An MPU sensor was used to acquire data at different engine loads for this research. The MPU sensor was mounted on the camshaft, as shown in Figure 4, and the specifications of the engine under test are shown in Table 1. The sensor recorded the motion of the camshaft at a sampling rate of 10 MHz. The MPU sensor converts the rotation of the camshaft into a time waveform. The primary signal extracted by the pickup sensor is shown in Figure 5, which is one revolution of the camshaft defined as a single period. A single period contains a total of 199 teeth and one missing tooth which shows the 12 cylinders’ ignition behavior. Cylinders in each bank explode the fuel in the proposer sequence over one period.

To extract data under different engine loads, the engine was run at different levels of engine load. At each level of engine load, the MPU sensor data that represent the revolutions of camshaft are stored. We consider five classes of engine load, and each class represents different ranges of engine loads. The efficiency of an engine is best at a load between 70% and 100% [22]. The most common operating load of the engine used in the experiment has two levels, i.e., 100% and 75%. Therefore, Class 1 means a full engine load, i.e., 97–100%, and Class 2 has an engine load of 72% to 78%. As the engine starts, it goes from 0% to maximum load steadily; therefore, two other engine load levels with a margin of 3% were also considered. Hence, classes 1, 2, 3, and 4 represent engine loads at 98.5 ± 1.5%, 75 ± 3%, 50 ± 3%, and 25 ± 3%, respectively, while Class 5 contains all other engine load levels that are not included in the first four classes.

## 5. Sensor Signal Pre-Processing for Feature Extraction

**Figure 6:**
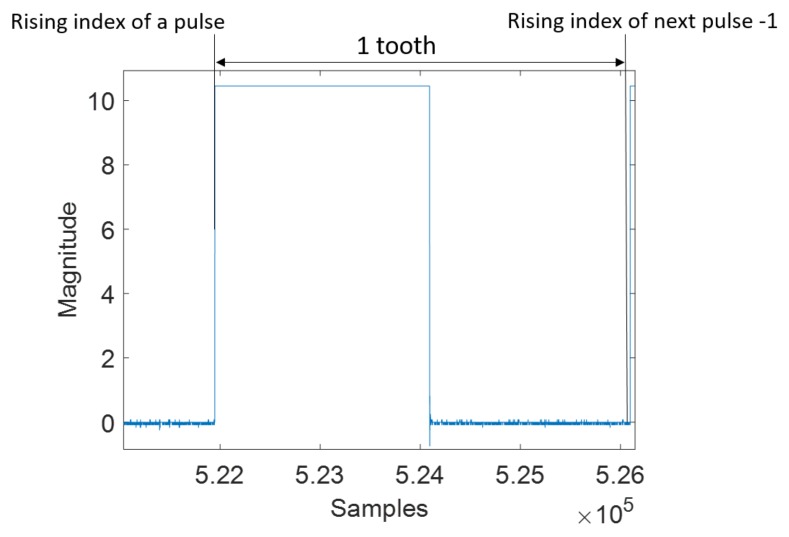
A single pulse of a pickup sensor data.

After data acquisition, we exploit the pickup sensor voltage signal to analyze the behavior of the combustion strokes in the firing cylinders of the engine. Since the crank angle at each cylinder’s ignition is known, the sensor signal is converted into a CAD signal. CAD is the unit used to measure the piston travel (position) during the firing sequence, and the CAD signal indicates the effect of the combustion stroke of each cylinder in the engine. Furthermore, the angle of rotation of the crankshaft measured from TDC is known as the crank angle, and when the piston is at TDC, the crank angle is 0 CAD. Each tooth in the sensor signal represents 3.6${}^{\circ }$ crank angle, and the number of data values (samples) in the 3.6${}^{\circ }$ crank angle represents the pulse interval (length) of a tooth of the sensor signal. Figure 6 shows a single tooth in the pickup sensor signal. The data values in each 3.6${}^{\circ }$ crank angle represent the CAD signal.

**Figure 7:**
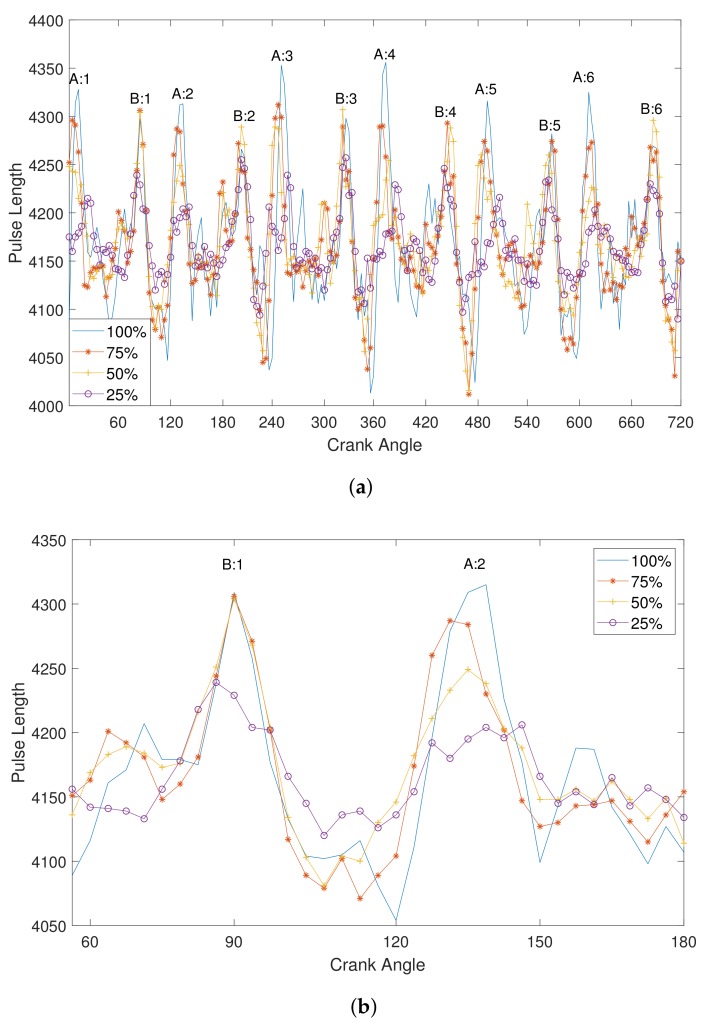
CAD signals of different engine loads: (**a**) full CAD signal represents the combustion strokes of 12 cylinders, and (**b**) part of the CAD signal represents combustion strokes of two cylinders.

The CAD signals of the different loads shown in Figure 7 correspond to two revolutions of the crankshaft, which is equal to 720 CAD. Engine cylinders connected to the camshaft sequentially ignite fuel, one by one, and a single revolution of the camshaft completes after all 12 cylinders ignite the fuel. The CAD signal is a row vector of 200 elements, since there is a total of 199 teeth in a single period and one missing tooth. Each element of the CAD signal represents the number of samples in one tooth of the MPU sensor data. The CAD signal corresponds to one revolution of the camshaft, which shows the effect from combustion strokes of the firing cylinders at a particular engine load. Figure 7a shows one revolution of the camshaft at different engine loads in the form of CAD signals. Each cylinder of the cylinder banks is fired within 720 CAD. The effect of the combustion strokes of firing cylinders is different for each engine load, and the ignition behavior of each cylinder at different loads can be observed in the CAD signals in Figure 7a. Figure 7b represents only the combustion stroke of one cylinder from each cylinder bank, and the difference in combustion strokes of firing cylinders under each engine load can be clearly observed. The CAD signal of a higher engine load has a higher magnitude and is more prominent during the firing of the cylinders. As we can see in Figure 7b, the ignition behavior of cylinder 2 of bank A (A:2) at a 25% load is not as prominent as a 100% load. The ignition behavior of the cylinders during a combustion stroke at higher engine loads, i.e., 100% and 75%, is more similar at lower loads. Also, the magnitude of a 100% load CAD signal is higher than other engine loads due to inertia. Another notable difference in CAD signals under engine loads is that firing of cylinders is not synchronized for all engine loads. For example, in Figure 7a, the crank angle for firing cylinders A:3, A:5, and A:6 at a 25% load is different from a 100% load. Hence, the ignition behavior of the firing cylinders of the engine can be analyzed under different engine loads by using CAD signals. Therefore, the CAD signal is considered a feature for classifying engine loads, and is input to the designed classifier.

## 6. ANN Classifier

Artificial neural networks are a set of algorithms that try to simulate the structure and functionalities of biological neural networks [23]. An ANN acquires information through a learning process, and performs specific tasks, such as pattern recognition and classification. There are different types of ANN, and each type of neural network is specific to a particular task. A feedforward neural network, also called an MLPNN, is used for supervised learning. It consists of several neurons-like processing units, organized in layers called hidden layers. Every neuron in a hidden layer is connected to all neurons of the previous layer. Each connection of the neurons with the adjacent layer’s neurons represents weight. The weight of each connection encodes the information of the network. In the feedforward neural network, data entered at the input layer is processed in the hidden layers and arrives at the output layer. There is no feedback between layers, and information flows in only one direction with no back-loop; that is why they are called feedforward neural networks. In general, there can be more than one hidden layer, and the number of neurons in each layer can vary.

### 6.1. Architecture of ANN

We used a feedforward neural network as a classifier to classify engine loads. To design the feedforward neural network, many parameters of the network are determined. The parameters of the network involve the number of neurons in each layer, as well as the number of hidden layers. The design parameters of the neural network can be selected by trial and error [24]. There are two approaches, known as the constructive approach and the destructive approach, to finding the optimal network size [25]. We used a constructive approach to tuning the network size. In the constructive approach, we start with a small network and gradually add units or connections to improve the performance of the network [25]. Different networks were built with different numbers of neurons in the hidden layer and were tested for minimum error. The number of hidden layer neurons varied between 5 and 50. The network with 10 neurons in a hidden layer turned out to have the minimum error. The designed classifier is a shallow feedforward neural network, which includes one hidden layer between input and output layers. The input layer contains 200 neurons, as the CAD signals are row vectors of 200 elements, and the output layer has five neurons because the designed network has five output classes.

### 6.2. Training of the Designed Network

**Figure 8:**
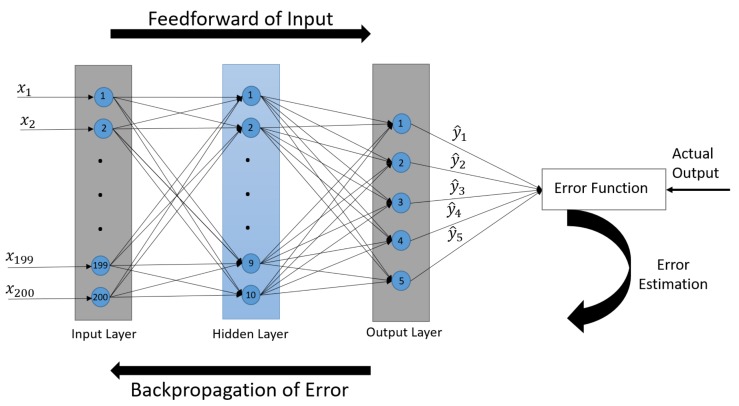
Structure of the designed classifier with a backpropagation method.

The operation of the feedforward neural network can be divided into two phases: learning and classification. In the learning phase, the input signal is forwarded to calculate the propagated error, and then weights in the network are updated. The designed classifier uses a supervised learning technique in which algorithms learn from labeled data [26]. A scaled conjugate gradient (SCG) algorithm [27] along with a backpropagation algorithm are used to train the feedforward neural network. Instead of estimating step size using a line-search technique, the SCG algorithm used the Levenberg-Marquardt algorithm combined with a conjugate gradient approach. The backpropagation algorithm calculates the error, and propagates it back through the network, and after that, weights are adjusted to reduce the value of the error function. To adjust the weights, the SCG algorithm updates them in the direction in which the performance function decreases most rapidly, i.e., the negative of the gradient, in each iteration. This decreases the value of the error function, which is the main objective to train a neural network. The hidden layer uses a rectified linear unit (ReLu) activation function whereas a SoftMax activation function is used for the output layer because it is used for multi-class classification. The cross-entropy error function is used to measure the performance of the designed classifier, and training the network involves minimizing cross-entropy to get high a classification accuracy. After training the network, weights are fixed in the classification phase. The structure of the designed feedforward neural network with backpropagation is shown in Figure 8.

## 7. Experimental Results

MATLAB software was used to implement the ANN for classification. The SCG algorithm is used with the backpropagation algorithm to train the designed network, which reduces errors between output and target values. The trained network should be able to classify engine loads samples into any one of the five loads, as depicted in Table 2. After training the network, we evaluated the performance of the network on unseen data that will access a generalization of the trained network [25].

### 7.1. ANN Training Performance

The CAD signal set, which is input data to the designed network, was randomly divided into three sets: 70% for training, 15% for validation, and 15% for testing. A total of 2450 input samples were used, out of which 1714 samples were for training, 368 for validation, and the remaining 368 samples for testing. Training of the network stops when generalization stops improving, i.e., training and validation errors stop decreasing.

**Figure 9:**
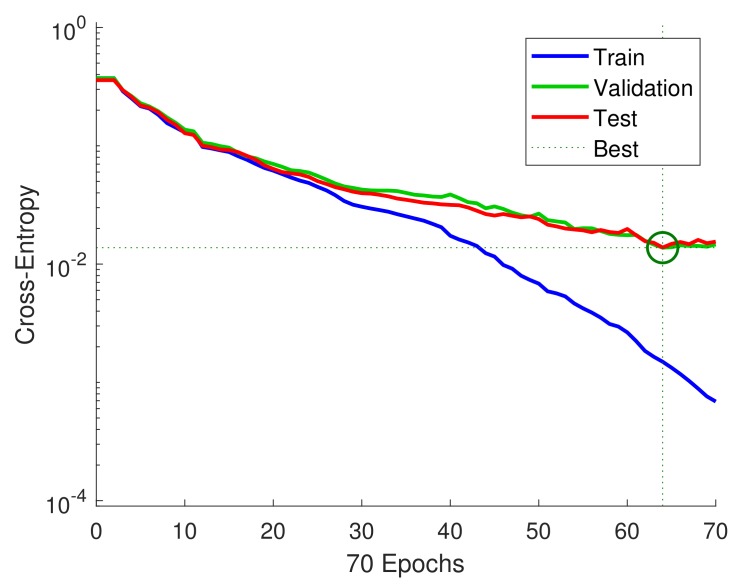
Training performance of the designed Feedforward neural network.

The training performance of the designed classifier is shown in Figure 9. The training of the classifier stopped after 70 steps, when cross-entropy reached a minimum value, as depicted in Figure 9. Cross-entropy measures the difference between actual output and predicted output. Minimizing the cross-entropy results in good classification. Cross-entropy of the designed classifier is close to zero for each set, and the best validation performance was 0.0136764 at epoch 64.

**Figure 10:**
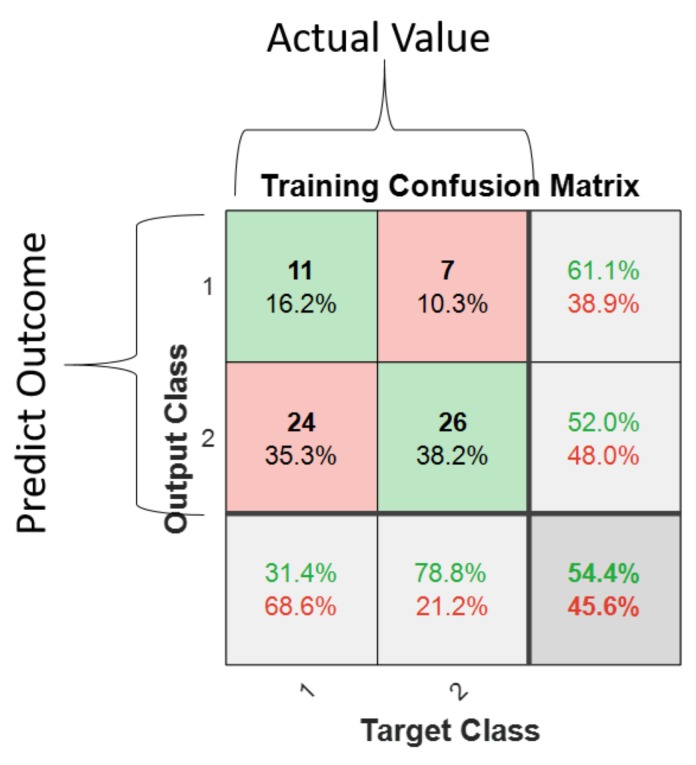
An example of confusion matrix.

**Figure 11:**
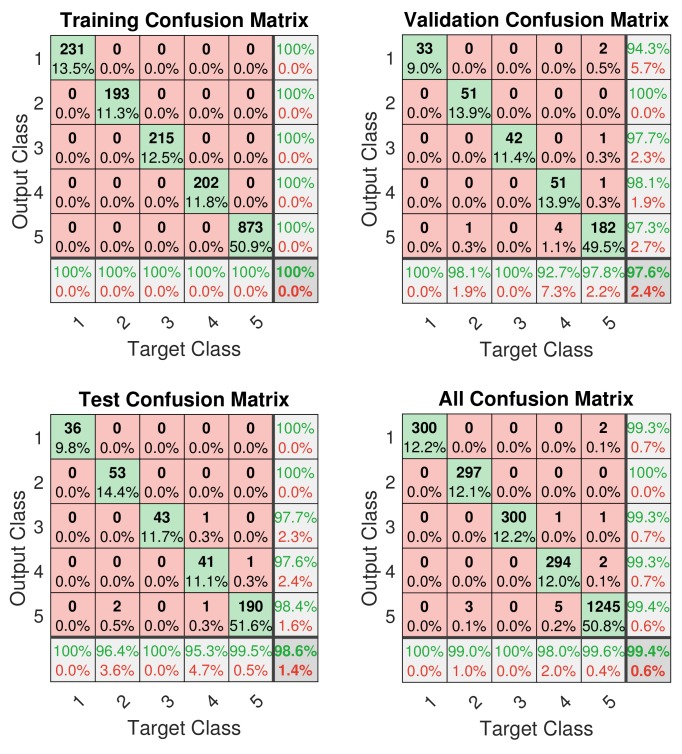
Confusion matrix of the designed classifier.

A confusion matrix is used to describe the performance of the classification systems, which contains information about actual and predicted classifications [28]. Considering the confusion matrix in Figure 10, there are two possible predicted classes: 1 and 2. Each row of the matrix represents the instances in a predicted class, while each column represents the instances in an actual class. Numerical and percentage values in the confusion matrix are the number of samples of a particular class and the percentage of the input samples, respectively. The first element of the first row shows the correctly classified input samples that belong to Class 1, while the second element of the first row shows the wrongly classified input samples that actually belong to Class 2, but were classified as Class 1. Similarly, the first element of the second row shows the wrongly classified input samples that actually belong to Class 2, but were classified as Class 1, and the second element of the second row shows the correctly classified input samples that belong to Class 2. For example, in Figure 11, values in the first row and second column show that seven samples, or 10.3% of the total input samples from Class 2, were wrongly classified as Class 1.

The last element of the first two rows has two percentage values. The first and the second percentage values show the percentages of the correctly and wrongly classified samples, respectively. The percentage values in the last element of the first column show the percentage of correctly and wrongly classified samples from Class 1 samples, respectively. Similarly, the percentage values in the last element of the second column show the percentage of correctly and wrongly classified samples from Class 2 samples, respectively. The percentage values in the last element of the first column show the percentage of correctly and wrongly classified samples from Class 1 samples, respectively. Similarly, the percentage values in the last element of the second column show the percentage of correctly and wrongly classified samples from Class 2 samples, respectively.

The confusion matrices of the designed classifier are shown in Figure 11, which describe the performance of the trained network with input data. The training confusion matrix in Figure 11 shows 100% classification accuracy during the training process. Furthermore, validation and test confusion matrices show that nine samples during validation and five samples from the test set are wrongly labeled. Overall, 300 samples from classes 1 to 4, and 1250 samples from class 5 are fed into network, and the network labels 2436 samples, i.e., 99.4%, with the correct class.

Considering the results of the designed feedforward neural network in Figure 9 and Figure 11, the network shows high classification accuracy on all three data sets. Some samples of Class 5 that are close to engine loads of classes 1–4 can possibly be misclassified. However, only five samples out of 600 for Class 5 were incorrectly classified. Therefore, the designed ANN can classify the different engine loads with an accuracy close to 100%.

### 7.2. Real-Time Evaluation of the Trained Network on Unseen Data

**Figure 12:**
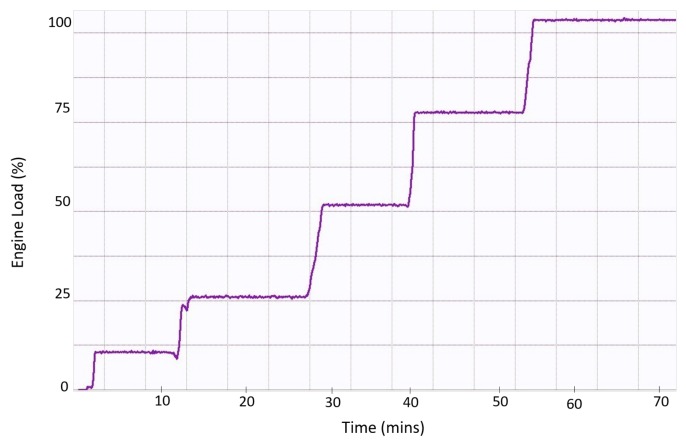
Real-time engine load information.

To evaluate the performance of the trained classifier on unseen data, we provided to the network data sets that are independent of the data set used to train the network. The data set are derived from the real-time measured data that represents the revolution of the camshaft of the engine under particular engine load. Figure 12 depicts the real-time engine load information measured from a real operating engine in which engine loads reached 100% from 0% in 70 min. In this duration, many samples are recorded under each engine load by using the MPU sensor. We used the samples from the real-time engine load information, which is depicted in Figure 12, for the unseen data set to evaluate the performance of the trained network solution of the camshaft at particular engine loads. A total of 5 data sets are fed into the trained classifier. Each data set consists of 200 samples and is belong to one of the engine load classes.

The classification accuracy for the 5 data sets are given in Table 3. The classifier labeled 97.7% samples of these data sets with the correct class. The classification accuracy on these unseen data sets shows that the trained network learned with generalize to new data and avoid overfitting. Also, the trained network can be used to classify engine loads in real time based on one revolution of a camshaft (83.3 ms). Hence, the designed classifier can tell the exact load condition, which helps to optimize the parameters to increase the efficiency of the engine, and can furthermore be used for the safety of the engine.

### 7.3. Comparison of the Designed ANN and SVM

To validate the performance of the designed classifier, we compared the designed network performance with another supervised learning classifier, i.e., the SVM classifier. The SVM was combined with an error-correcting output codes (ECOC) model for multi-class engine load classification. The ECOC method is commonly used to solve multi-class classification problems [29]. The SVM’s binary learners are used to train a multi-class ECOC model, and the same algorithm, which is described in Figure 3, is used to classify engine loads using the SVM classifier. The same data set was used for training the SVM learning model, and the CAD signal is considered to be feature input for the SVM classifier.

Table 4 shows a comparison of the ANN and the SVM performance for multi-class engine load classification. The training error of the ANN classifier is slightly higher than the SVM classifier, whereas generalization error of the SVM classifier is much greater than the designed ANN classifier. This shows that the ANN learns to predict new data with higher accuracy and avoids overfitting. Hence, the ANN is useful in accounting for nonlinear behaviors of the engine, although other machine learning models can also be used to classify engine loads. Another advantage of an ANN over other learning models is that if an ANN is trained on one task, its parameters, i.e., the number of hidden neurons, the learning rate, etc., can be used as a good initializer for similar tasks.

## 8. Conclusions

In this paper, we proposed an engine load classifier for a V12 marine diesel engine using an artificial neural network. Real measured data were used to verify the classification performance of the designed network. Supervised learning, one of the machine learning technique for classification, was used along with the ANN.

Engine load classification helps to optimize the engine control parameters, which increases the efficiency of the engine. The combustion strokes of firing cylinders in an engine are different under differing engine loads, which leads to adjusting the combustion parameters based on engine load. Furthermore, engine load classification can be used as a tool in a marine engine control system to increase the efficiency and safety of the engine. A magnetic pickup sensor was used to measure the rotation of a tooth gear under different engine loads. To extract the behavior from internal combustion of an engine under a particular engine load, sensor signals were converted into CAD signals. The shape of CAD signals differs for each engine load. Hence, a CAD signal that represents one revolution of the camshaft is considered a feature characteristic for classifying engine loads. A feedforward neural network was designed, and a backpropagation algorithm was used to train the network for supervised classification. The network classified engine loads using the CAD signals of each load as a feature vector.

To verify the performance of the designed neural network, we considered five classes of engine load (98.5 ± 1.5%, 75 ± 3%, 50 ± 3%, 25 ± 3%, and other loads) in the V12 marine diesel engine. The designed network classified engine loads using real measured data at a classification accuracy of 99.4%. We also compared the results of the designed ANN classifier with the SVM classifier. The SVM showed low training error, but the proposed ANN showed better generalization to unseen data. The proposed engine load classifier can be implemented for the same type of engine with different numbers of cylinders.

## Figures and Tables

**Table 1:** Test Engine Specifications.

Manufacturer	Hyundai Heavy Industries
Engine type	V engine
Related power	7200 KW
Rotational speed	720 rpm
Type fuel	Diesel
Firing order	Bank A: 1-2-3-4-5-6 Bank B: 1-2-3-4-5-6

**Table 2:** Analyzed Loads.

Classes	Target Output	Engine Load
1	1 0 0 0 0	98% ± 1.5%
2	0 1 0 0 0	75% ± 3%
3	0 0 1 0 0	50% ± 3%
4	0 0 1 0 0	25% ± 3%
5	0 0 0 0 1	others

**Table 3:** Classification accuracy of the trained network on other unseen data sets.

Data Set	Engine Loads	Input Samples	Classification Accuracy
1	Class 1	200	100%
2	Class 2	200	95.0%
3	Class 3	200	97.0%
4	Class 4	200	98.5%
5	Class 5	200	98.0%
Total		1000	97.7%

**Table 4:** Comparison of classification errors by the ANN and the SVM.

Learning Model	Training Error [%]	Generalization Error [%]
Feedforward NN	0.6	2.3
SVM	0.4	6.65
